# Slit Lamp Report Generation and Question Answering: Development and Validation of a Multimodal Transformer Model with Large Language Model Integration

**DOI:** 10.2196/54047

**Published:** 2024-12-30

**Authors:** Ziwei Zhao, Weiyi Zhang, Xiaolan Chen, Fan Song, James Gunasegaram, Wenyong Huang, Danli Shi, Mingguang He, Na Liu

**Affiliations:** 1 School of Optometry The Hong Kong Polytechnic University Hong Kong China; 2 Monash University Victoria Australia; 3 Zhongshan Ophthalmic Center Sun Yat-sen University Guangzhou China; 4 Research Centre for SHARP Vision The Hong Kong Polytechnic University Hong Kong China; 5 Centre for Eye and Vision Research (CEVR) Hong Kong China; 6 Guangzhou Cadre and Talent Health Management Center Guangzhou China

**Keywords:** large language model, slit lamp, medical report generation, question answering

## Abstract

**Background:**

Large language models have shown remarkable efficacy in various medical research and clinical applications. However, their skills in medical image recognition and subsequent report generation or question answering (QA) remain limited.

**Objective:**

We aim to finetune a multimodal, transformer-based model for generating medical reports from slit lamp images and develop a QA system using Llama2. We term this entire process slit lamp–GPT.

**Methods:**

Our research used a dataset of 25,051 slit lamp images from 3409 participants, paired with their corresponding physician-created medical reports. We used these data, split into training, validation, and test sets, to finetune the Bootstrapping Language-Image Pre-training framework toward report generation. The generated text reports and human-posed questions were then input into Llama2 for subsequent QA. We evaluated performance using qualitative metrics (including BLEU [bilingual evaluation understudy], CIDEr [consensus-based image description evaluation], ROUGE-L [Recall-Oriented Understudy for Gisting Evaluation—Longest Common Subsequence], SPICE [Semantic Propositional Image Caption Evaluation], accuracy, sensitivity, specificity, precision, and *F*_1_-score) and the subjective assessments of two experienced ophthalmologists on a 1-3 scale (1 referring to high quality).

**Results:**

We identified 50 conditions related to diseases or postoperative complications through keyword matching in initial reports. The refined slit lamp–GPT model demonstrated BLEU scores (1-4) of 0.67, 0.66, 0.65, and 0.65, respectively, with a CIDEr score of 3.24, a ROUGE (Recall-Oriented Understudy for Gisting Evaluation) score of 0.61, and a Semantic Propositional Image Caption Evaluation score of 0.37. The most frequently identified conditions were cataracts (22.95%), age-related cataracts (22.03%), and conjunctival concretion (13.13%). Disease classification metrics demonstrated an overall accuracy of 0.82 and an *F*_1_-score of 0.64, with high accuracies (≥0.9) observed for intraocular lens, conjunctivitis, and chronic conjunctivitis, and high *F*_1_-scores (≥0.9) observed for cataract and age-related cataract. For both report generation and QA components, the two evaluating ophthalmologists reached substantial agreement, with κ scores between 0.71 and 0.84. In assessing 100 generated reports, they awarded scores of 1.36 for both completeness and correctness; 64% (64/100) were considered “entirely good,” and 93% (93/100) were “acceptable.” In the evaluation of 300 generated answers to questions, the scores were 1.33 for completeness, 1.14 for correctness, and 1.15 for possible harm, with 66.3% (199/300) rated as “entirely good” and 91.3% (274/300) as “acceptable.”

**Conclusions:**

This study introduces the slit lamp–GPT model for report generation and subsequent QA, highlighting the potential of large language models to assist ophthalmologists and patients.

## Introduction

The slit lamp, a cornerstone in ophthalmology, allows for detailed examination of the eye’s anterior segment [1]. Using an illuminated, narrow beam, this noninvasive method facilitates the evaluation of abnormalities by depth and size. While instrumental in diagnosing common eye diseases such as keratitis, conjunctivitis, conjunctival concretions, and cataracts, interpreting slit lamp results can be challenging for primary care physicians due to the need for specialized training. This can result in overlooked abnormalities or misdiagnosis. Furthermore, ophthalmologists are tasked with interpreting, documenting, and effectively communicating these results to patients, a time and effort-intensive process. The scarcity of experienced ophthalmologists, particularly in rural areas, further exacerbates the situation [2].

Artificial intelligence (AI) and large language models (LLMs) have made significant strides in the medical field, enhancing the capabilities of health care professionals in interpreting, and analyzing medical imagery. For instance, AI has been instrumental in advancing the analysis of x-rays [3], magnetic resonance images [4], ultrasounds [5], and dermatological images [6]. Generative pretrained transformers (GPT) models such as ChatGPT [7] and Llama2 [8], have showcased remarkable capabilities in problem-solving scenarios across a spectrum of medical applications. These AI models are instrumental in streamlining clinical documentation [9], refining patient communication [10], aiding administrative tasks [11], enriching textual data [12], and bolstering evidence-based decision-making [13]. Their versatility extends to comprehensive patient assessments [14], precise disease diagnostics [15], informed treatment proposals [16], meticulous medical writing [17], innovative teaching methodologies [18], and robust question answering (QA) systems [19], embodying a multifaceted impact on the health care industry.

Deep learning strategies currently used to transform images into high-quality features include convolutional neural networks (CNNs), recurrent neural networks (RNNs), transformer networks, and their variants such as long short-term memory (LSTM) and gated recurrent units (GRUs). CNNs are often combined with other networks such as RNNs to generate text [20]. RNNs and their variants, recognized for their prowess in handling sequential data, account for element dependencies within sequences. Despite their effectiveness, RNNs face challenges with extended sequences and potential gradient issues which are mitigated by long short-term memories and GRUs through a gate mechanism. Transformer networks, proposed in 2017, use self-attention mechanisms to manage long sequences and parallel computations, thus boasting swift training speed at the cost of substantial computational resources [21]. Bootstrapping Language-Image Pre-training (BLIP), a hybrid approach leveraging transformer networks’ architecture and amalgamating natural language processing and computer vision, enhances model performance via pretraining. BLIP’s principal strength lies in its multimodal capacity to concurrently handle image and text data, allowing it to excel in specific tasks such as image description generation.

In the specific context of slit lamp imaging augmented with AI, research has primarily concentrated on individual disease detection and grading, such as in the case of cataracts [22,23], pterygium [24], and infectious keratitis [25]. However, there is a noticeable lack of a unified system that uses slit lamp images for the generation of systematic anterior segment reports and QA. While the advent of OpenAI’s GPT-4V offered the possibility of image-based AI medical dialogue, its direct clinical application has been limited by inaccuracies and the generation of unreliable information, which was termed “hallucinations” [26,27]. Additionally, due to its closed-source nature, there is a constraint on the fine-tuning ability, which is paramount for medical applications. In response to this, our study has used Llama2, an open-source model, to harness the anticipated benefits of a specialized LLM tool that ensures enhanced control and reliability in the subsequent QA scenarios. Based on our experience in ophthalmic QA tasks and LLMs, including fundus fluorescein angiography and indocyanine green angiography QA [12,28,29] we aim to extend these methodologies to slit lamp imaging by developing a novel slit lamp–GPT system, using BLIP and LLMs specifically tailored for ophthalmology, with dual objectives: to generate reports and to facilitate QA.

## Methods

### Overview

**Figure 1:**
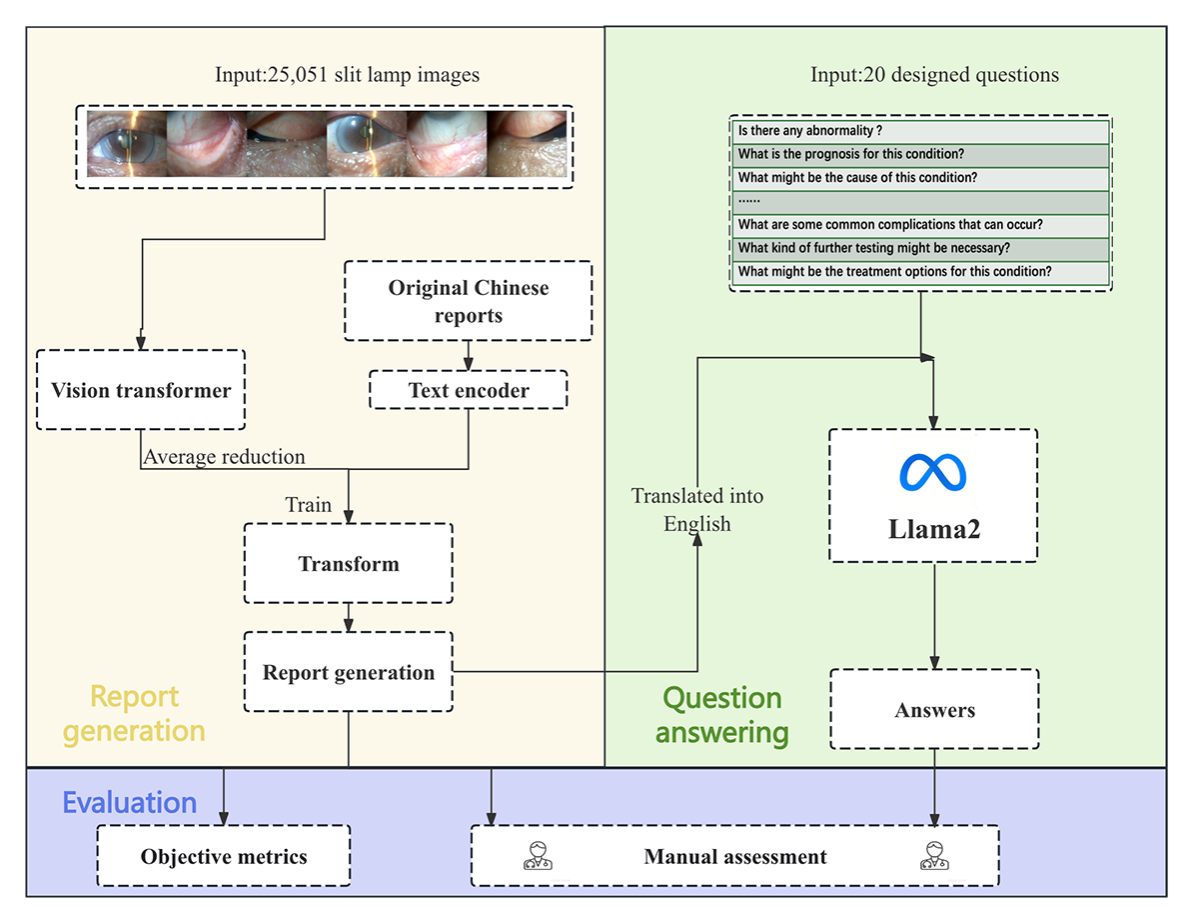
Flow diagram of this study.

The flow of our study is outlined in Figure 1.

### Dataset

We collected data from a Chinese physical examination center for this retrospective study, which included both essential clinical information and annual slit lamp images. We included slit lamp photographs with corresponding medical reports, excluding any of inadequate quality. This study used data collected from a previous study [30], all participants’ information was deidentified per the Declaration of Helsinki’s guidelines. All slit lamp images, captured via a Haag-Streit BQ-900 at a 2048×1536-pixel resolution, included at least 4 images per participant showcasing the pupil, upper eyelid, and lower eyelid. Initial reports, written by ophthalmologists in Chinese, contained disease diagnoses, recommendations, or detailed descriptions of ocular signs. A subset of representative reports from the dataset was selected for translation into English to form a bilingual dataset.

### Model Construction

Similar to other studies [28,29], we initially trained and tested the BLIP [28] network for report generation. Subsequently, the generated reports from the test set were input into Llama2 for QA validation, further evaluating the quality and practicality of the reports.

During the report generation phase, we used the BLIP framework, a multimodal transformer model skilled at aligning visual interpretation with text generation. The model filtered out noisy data during training and generated slit lamp reports from paired images and text inputs. Our design incorporated a vision transformer [31] and BERT [32] as the image and language encoder and decoder, respectively. The vision transformer converts an image into encoded patch sequences, while BERT, trained on extensive unlabeled text data, enables deep contextualized representation learning. The pretrained BLIP model was fine-tuned using slit lamp images and associated reports, with each case providing at least four images during training, resized to 224×224 pixels. We applied the AdamW optimizer (the University of Freiburg), using an initial learning rate of 0.00002, a weight decay of 0.05, and a cosine learning rate schedule, across 50 epochs on one NVIDIA Tesla V100 GPU (NVIDIA Corp). The model with the highest BLEU1 (bilingual evaluation understudy) score (detailed in the performance evaluation part) on the validation set was selected for testing.

For the question and answering phase, we created a question set related to slit lamp examination and reporting based on prior studies [33] and our clinical expertise. These questions, along with the corresponding reports, were seamlessly input into the Llama2 model. This integration allowed for QA without the need for fine-tuning, while enhancing the interpretation of the generated reports. The process involved instructing the model using a specific prompt: “Answer based on: [slit lamp report content here].”

### Performance Evaluation

We used both language-based and disease classification metrics for quantitative evaluations of report quality, supplemented by manual assessments for report generation and QA.

For language-based metrics, we used BLEU [34], CIDEr [35], ROUGE-L [36]), and Semantic Propositional Image Caption Evaluation [37], each with its strengths. However, traditional language metrics may be less dependable for medical conditions due to the infrequent occurrence of disease-related keywords in reports. To address this, we introduced a classification evaluation procedure that used a manually curated dictionary to identify disease-related conditions or postoperative statuses from both original and generated reports. Disease classification metrics, such as specificity, accuracy, precision, sensitivity, and the *F*_1_-score, provided a comprehensive performance review of the model.

Considering the complexity of medical terminology and the potential harm of inaccurate reporting, manual assessment remains crucial. For report generation, 100 test set cases were randomly selected and independently evaluated by 2 ophthalmologists (ZZ and FS) using a 3-point scale, focusing on “completeness” (how well the generated reports matched the ground truth conditions) and “correctness” (the accuracy of diagnosis and condition descriptions). Scores ranged from 1 (excellent) to 3 (poor), with 2 representing an acceptable rating. The final score was the average of the scores from the 2 evaluators. For QA, 20 prepared human-posed questions and the translated report were put into Llama2 to generate answers, which were evaluated based on “completeness,” “correctness,” and “possible harm.” Scores ranged from 1 (recommendable to patients) to 3 (not recommendable for patients), with 2 indicating that minor adjustments could make the answer suitable for recommendation. The average score was also used as the final score. For detailed scoring criteria in these 2 sections, refer to Table S1 in Multimedia Appendix 1.

### Ethical Considerations

This study used data collected from a previous study [30]. All patient data were anonymized and de-identified following the Declaration of Helsinki. Individual consent was waived due to the retrospective nature and the thorough anonymization process of the study. The Institutional Review Board of the Hong Kong Polytechnic University approved the study (HSEARS20240301004).

## Results

### Data

Our final dataset includes 25,051 slit-lamp images and 3409 reports. Most images (12,496, 49.89%) focus on the cornea, with 32.74% (n=8202) on the upper eyelid and 17.38% (n=4353) on the lower eyelid. The median age of participants is 65, with an IQR of 60 to 72 years, and the majority (2009/3409, 58.93%) are male. The demographics and image types are similar across all sets.

The distribution of images across years is as follows: 1257 (5.02%) from 2013, 12,206 (48.72%) from 2015, and 11,588 (46.26%) from 2016. The 2013 and 2015 images form the training set, while the 2016 images are partitioned evenly into validation and testing sets. There were no significant differences in demographic characteristics and positioning type between these datasets. Table 1 provides a comprehensive overview of the dataset characteristics.

**Table 1:** Slit lamp images: dataset characteristics.

					Total		Train	Validation		Test		*P* value	
**Participants**													
	Number				3409		1846	781		782			
	Age, median (Q1^a^, Q3^b^)				65.46 (60.52, 72.47)		65.31 (60.12, 72.13)	62.04 (59.58, 65.9)		71.04 (65.47, 77.03)		<.001	
	**Sex, n (%)**												.002
			Male	2009 (58.93)		1101 (59.64)		420 (53.78)	488 (62.4)				
			Female	1400 (41.07)		745 (40.36)		361 (46.22)	294 (37.6)				
**Slit lamp images**													
	Number				25,051		13,463	5987		5601			
	**Position, n (%)**												.002
		Upper eyelid		8202 (32.74)		4315 (32.05)		2046 (34.2)	1841 (32.9)				
		Lower eyelid		4353 (17.38)		2423 (18)		951 (15.9)	979 (17.5)				
		Cornea		12,496 (49.89)		6725 (49.95)		2990 (49.9)	2781 (49.7)				

^a^Q1: first quartile.

^b^Q3: third quartile.

We used a custom dictionary to extract diagnoses and physical signs by keyword matching from the Chinese reports. We identified 50 conditions, including age-related cataracts (478/2170, 22.03%), cataracts (498/2170, 22.95%), conjunctival concretion (285/2170, 13.13%), after intraocular lens implantation (151/2170, 6.96%), pterygium (144/2170, 6.64%), conjunctivitis (97/2170, 4.47%), chronic conjunctivitis (93/2170, 4.29%), and other eye conditions with lower proportions. This led to 1377 Chinese reports primarily featuring diagnostic terms or descriptions of ocular signs.

### Quantitative Model Performance

Language-based metrics are provided in Table 2, with BLEU (1-4) scores (0.67, 0.66, 0.65, and 0.65) indicating good lexical accuracy and a ROUGE-L score of 0.61 highlighting effective content retention. The CIDEr score of 3.24 reflects its ability to align closely with human judgment on sentence quality, while a SPICE score of 0.37 demonstrates moderate success in capturing complex semantic relationships. For disease classification metrics (see Table 3), our model achieved a weighted accuracy of 0.82 and a weighted *F*_1_-score of 0.64. However, performance varied across diseases. It was highly accurate (≥0.9) for conditions of intraocular lens, conjunctivitis, and chronic conjunctivitis, and had high *F*_1_-scores (≥0.9) for cataracts and age-related cataracts. The model demonstrated excellent accuracy for positive cases of cataracts and age-related cataracts. Despite high accuracy, specificity, and precision for postoperative intraocular lens implantation, sensitivity was relatively low: a clinically acceptable trade-off. However, for conjunctival concretions, conjunctivitis, and chronic conjunctivitis, the model’s overall predictive capacity fell short.

**Table 2:** Language-based metrics of report generation in the test set (5601 images from 782 participants).

BLEU_1^a^	BLEU_2^a^	BLEU_3^a^	BLEU_4^a^	CIDEr^b^	ROUGE^c^	SPICE^d^
0.67	0.66	0.65	0.65	3.24	0.61	0.37

^a^BLEU: bilingual evaluation understudy.

^b^CIDEr: consensus-based image description evaluation.

^c^ROUGE: Recall-Oriented Understudy for Gisting Evaluation.

^d^SPICE: Semantic Propositional Image Caption Evaluation.

**Table 3:** Disease classification metrics of report generation in the test set.

Condition	Specificity	Accuracy	Precision	Sensitivity	*F*_1_-score
Age-related cataract	0.6	0.79	0.9	0.8	0.84
Cataract	0.58	0.78	0.9	0.79	0.84
After intraocular lens implantation	0.93	0.94	0.96	0.48	0.64
Conjunctival concretion	0.83	0.7	0.37	0.44	0.4
Chronic conjunctivitis	0.92	0.9	0.34	0.15	0.2
Conjunctivitis	0.92	0.9	0.34	0.14	0.2

### Qualitative Model Performance

#### Overview

The score distribution is depicted in Figure S1 in Multimedia Appendix 2.

#### Report Generation

Two ophthalmologists scored the model highly for completeness (mean 1.36, SD 0.61, κ=0.84) and correctness (mean 1.36, SD 0.59, κ=0.72). Reports that received a score of 1 for both completeness and correctness were defined as entirely good and constituted 64% (64/100) of the evaluated reports. Reports that scored either 1 or 2 by both reviewers for both completeness and correctness were deemed acceptable, representing 93% (93/100) of the reports. These scores primarily corresponded to reports detailing specific conditions such as cataracts, age-related cataracts, and negative findings. However, 7% (7/100) of reports scored a 3, indicating deficiencies.

We discovered that lower scores were linked to issues such as limited sample sizes for specific diseases, difficulties in clearly identifying lesions, and challenges in interpreting diseases or signs from images due to the unique aspects of slit lamp photography. These complications were common in conditions such as xanthomas, trichiasis, after-glaucoma surgery, lagophthalmos, and some small conjunctival concretions. Additionally, images not focused on the cornea made it difficult to detect corneal lesions.

Through our hands-on evaluation, we noticed that the model sometimes added diagnoses that were not in the original reports but were still acceptable based on the images. For example, it sometimes diagnosed mild cataracts even when the images did not show apparent lens abnormalities. We considered these decisions acceptable when considering the challenge faced by an ophthalmologist in making a precise distinction based solely on images.

#### About QA

Our constructed questionnaires included 20 items, addressing a breadth of topics such as diagnosis, pathologic localization, severity grading, visual impairment, prognosis, associated complications, therapeutic recommendations, suggested further examinations, preventive advice, and scientific education pertinent to slit lamp examination (Table S2 in Multimedia Appendix 1).

We selectively curated 15 representative English reports on conditions including cataracts, conjunctival concretions, conjunctivitis, postintraocular lens implantation, and pterygium, as well as their mixed states. Each report contributed 20 questions, culminating in a total of 300 questions.

Our model scored well on completeness (1.33, κ=0.84), correctness (1.14, κ=0.71), and possible harm (1.15, κ=0.82). Similarly, QA responses that scored a 1 in completeness, correctness, and possible harm were defined as entirely good, representing 66.3% (199/300) of the QA responses. Responses scoring either 1 or 2 across these categories were considered acceptable, comprising 91.3% (274/300). Less than 9% (26/300) of the 300 questions scored a 3 in any category. These were typically related to reports focusing more on physical signs than diagnoses or conditions and statements about binocular intraocular lenses. Figure S2 in Multimedia Appendix 3 provides examples of generated answers with different scores.

## Discussion

### Principal Findings

**Figure 2:**
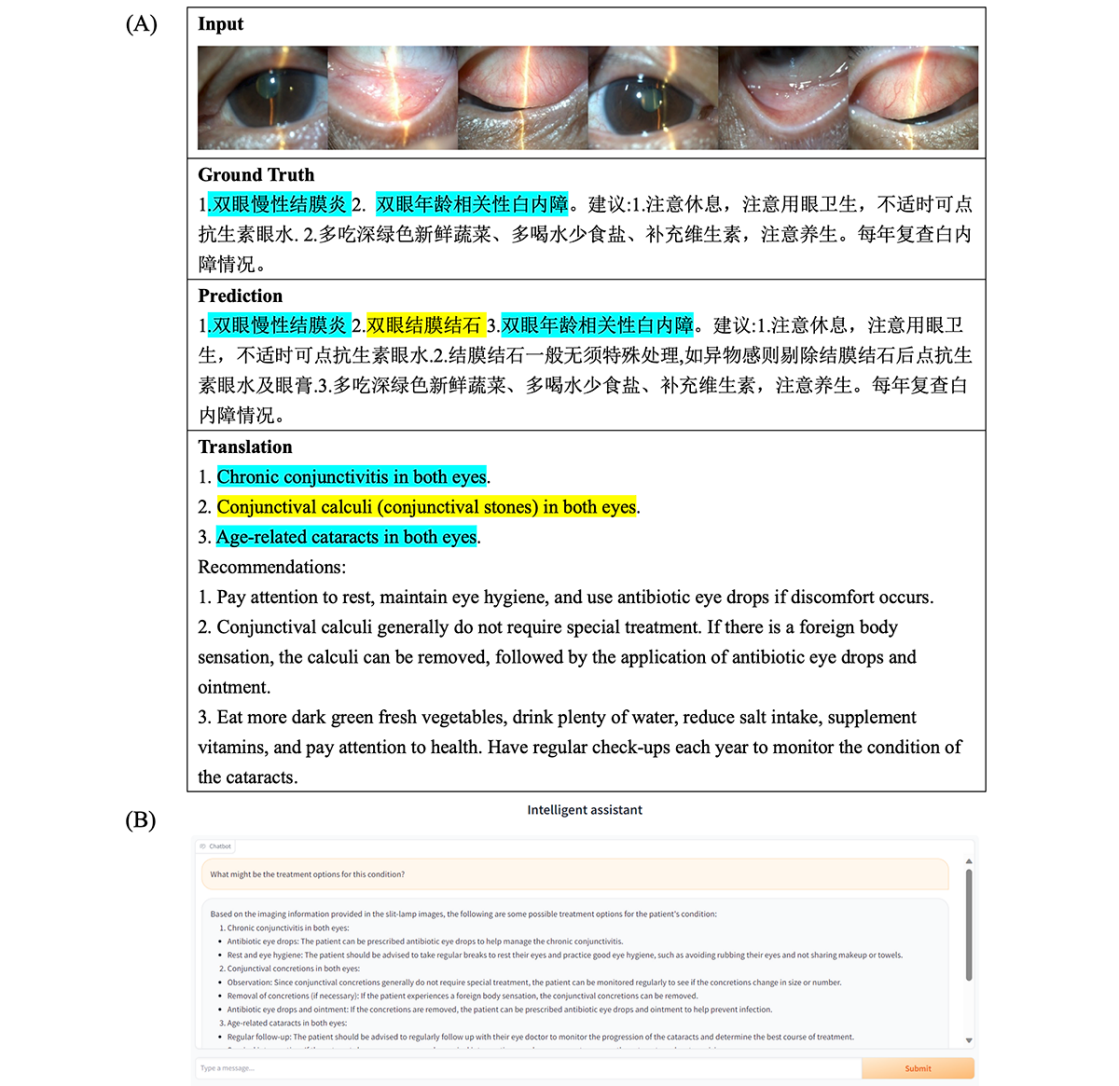
Demonstration of the question-answering system. (A) Input image, ground truth, and model prediction. (B) Question answering. Blue highlight: corresponds to accurate diagnosis matches. Yellow highlight: supplementary predicted information (not in the manual report but correct).

Our study introduces a novel method for analyzing slit lamp images through the integration of a multimodal transformer with an LLM. This approach has enabled the accurate identification of common anterior segment eye diseases and supports a QA system that directly addresses symptoms, diagnosis, and treatment options, as illustrated in Figure 2.

LLMs represent a breakthrough in AI with large knowledge bases and strong logical reasoning abilities. They have exhibited efficacy across various natural language processing tasks, including text generation, summarization, translation, and QA. However, in the medical realm, the quality of these answers warrants further scrutiny. Previous research has shown mixed results for the ability of LLMs to pass ophthalmology examinations. The study of Kung et al [38] indicates that ChatGPT can pass the United States Medical Licensing Examination without any specialized training or reinforcement. However, Thirunavukarasu’s [39] attempt to assess ChatGPT’s proficiency in the FRCOphth (Fellowship of the Royal College of Ophthalmologists) examination showed subpar performance, thereby underscoring the inability of LLMs to replace physicians in highly specialized fields. Conversely, in advising patients about symptoms or ongoing conditions—tasks less demanding of expertise—ChatGPT seems to demonstrate competence. Many patients turn to the internet for self-diagnosis before consulting a health care professional [40]. The use of LLMs for medical consultations can increase patient independence and potentially aid in accurate diagnosis. The release of GPT4V represents an innovative leap in the realm of LLM integration with computer vision, with promising prospects for extensive application in the medical field. Wu et al [26] assessed images from eight modalities across 17 human body systems and concluded that while GPT4V excels at identifying image modalities and anatomical structures, it encounters significant challenges in disease diagnosis and comprehensive report generation. In another study, we used a similar 1-3 evaluation scale to assess GPT4V’s performance on ophthalmology-related tasks, including image interpretation and QA [27]. The model performed best in analyzing slit lamp images; however, it only reached 42% (42/100) in accuracy, 38.5% (34.7/90) in usability, and 68.5% (61.7/90) in safety of the responses. These results are significantly lower than the “entirely good” rates we reported previously—64% (64/100) for report generation and 66.3% (199/300) for QA. This discrepancy underscores the need for models tailored to ophthalmology to ensure high-quality outcomes. To address this gap, we implemented an experimental model, slit lamp–GPT, harnessing the BLIP and Llama2 frameworks. This initiative represents merely the first step in a broader journey toward refining AI applications in ophthalmology.

The model demonstrated proficiency in identifying and reporting common anterior segment eye diseases within our dataset. However, its performance on rare conditions highlighted a critical area for improvement, suggesting that its effectiveness is closely tied to the diversity and representation of conditions in the training data. Per report generation, suboptimal performance was linked to specific diseases such as trichiasis, postglaucoma surgery complications, and corneal pathologies. Given our dataset’s origin in routine health examination data, these conditions were underrepresented, likely contributing to the poor performance. Another hypothesis considers the dynamic nature of slit lamp examinations in clinical settings, where ophthalmologists manually focus to obtain the best diagnostic view, a process not fully captured by static images. Instances of misdiagnosed keratitis, where images did not focus precisely on the cornea, support this assumption. Integrating our model with a broader spectrum of ophthalmic imaging techniques—such as indocyanine green angiography, fundus fluorescein angiography, ocular ultrasound, optical coherence tomography, and fundus photography—may enhance diagnostic alignment with actual clinical observations and further improve overall performance.

The current results suggest potential applicability in cataract screening, particularly in regions with a shortage of ophthalmologists. Previous studies have primarily focused on applying deep learning to the diagnosis and grading of cataracts, fundamentally using classification models. In contrast, our model is a natural language processing system capable of generating free-text reports. It not only provides descriptive insights but also achieves cataract classification accuracy similar to existing models [41,42]. Beyond this, our model could function as an educational tool for patients. In bustling eye clinics, patients may lack sufficient time to fully comprehend their examination reports and medical conditions. As demonstrated in this study, the slit lamp–GPT can provide patients with basic clinical explanations and recommendations concerning causes, abnormalities, treatment, and follow-up, indicating its potential to reduce medical consultation expenditure and bolster the use of remote health care services.

The manual evaluation suggests that slit lamp–GPT exhibits a promising capacity to assist participants with minimal risk. During the QA stage, 89.3% (268/300) of the responses were deemed completely harmless, surpassing the performance of GPT4V. However, the potential risks of using LLMs are yet to be thoroughly understood. A common problem with LLMs is that they sometimes generate inaccuracies and false statements, which are often referred to as “hallucinations” in the field [43]. These incorrect assertions can appear to be true, which could harm patients. This was reflected in our study, where the model sometimes created content. For example, the Llama2 model wrongly identified a binocular intraocular lens as a disease instead of a postoperative condition, creating the nonexistent “binocular intraocular lens syndrome.” This led to poor scores on the related 20 questions, highlighting the need for specialized fine-tuned LLM and knowledge-based generation [44]. Nonetheless, it is important to recognize that LLMs should serve as adjuncts or supplements in the clinical diagnosis and treatment process, not as fully trusted entities devoid of physician oversight. As LLM technology evolves, it is incumbent on stakeholders to collaboratively establish best practice standards to ensure patient safety.

### Limitations

This study has a few limitations. First, the dataset used is skewed, coming mainly from routine health checks of healthy people. The small sample size for certain diseases might affect the effectiveness of classification. Using datasets from high-quality outpatient clinics could lead to better results. Second, as with other language models, our model sometimes produces repetitive text, and the accuracy of the responses it generates can be inconsistent. At times, the model’s answers show logical errors. For instance, it diagnosed both a postintraocular lens implantation status and a senile cataract in the same eye. These issues might be addressed by incorporating expert knowledge and fine-tuning LLMs. There are also notable concerns about bias, as a single mistake in report generation can lead to multiple errors during the question-and-answer process. This highlights the need for further improvements to increase the accuracy and completeness of report generation. Lastly, creating a standardized manual evaluation process for these types of models is challenging [45,46]. This study was limited to slit lamp anterior segment images, indicating a need for future research to include diverse datasets. This will help evaluate the model’s applicability across various types of imaging.

### Conclusion

This research underscores the effectiveness and potential of using LLMs for slit lamp image report generation and QA tasks, showcasing their viability in ophthalmic medical image analysis.

## Appendix

Multimedia Appendix 1 Supplementary tables with data.

Multimedia Appendix 2 Distribution of scores in the manual assessment of report generation and question-answering. RG: report generation; QA: question answering.

Multimedia Appendix 3 Examples of generated answers with different scores. Red bold text indicates incorrect information.

## Abbreviations

AI: artificial intelligence
BLEU: bilingual evaluation understudy
BLIP: Bootstrapping Language-Image Pre-training
CIDEr: consensus-based image description evaluation
CNNs: convolutional neural networks
GRUs: gated recurrent units
LLM: large language model
QA: question answering
ROUGE-L: Recall-Oriented Understudy for Gisting Evaluation—Longest Common Subsequence
RNN: recurrent neural network
SPICE: Semantic Propositional Image Caption Evaluation
