# Supplementary material for: Slit Lamp Report Generation and Question Answering: Development and Validation of a Multimodal Transformer Model with Large Language Model Integration
Source: J Med Internet Res. 2024 Dec 30;26:e54047. doi: 10.2196/54047 (PMC11729784; doi:10.2196/54047)
Supplement: Multimedia Appendix 1 [file jmir_v26i1e54047_app1.docx]

**Supplementary Table 1**. The scoring criteria for manual assessment of report generation and question answering.

|  | Evaluation Index | Score | Interpretation |
| --- | --- | --- | --- |
| Report generation | Completeness | 1 | Identifies all diagnoses or diagnostic signs |
|  |  | 2 | Identifies key diagnosis or signs, but not all |
|  |  | 3 | Fails to identify key diagnosis |
|  | Correctness | 1 | Report is entirely accurate |
|  |  | 2 | Report has some inaccuracies |
|  |  | 3 | Report contains significant inaccuracies |
| Question Answering | Completeness/ | 1 | Appropriate for patient recommendation;  no modifications required |
|  | Correctness/ | 2 | Contains minor errors; Omit minor message；  requires fine-tuning prior to patient recommendation |
|  | Possible harm | 3 | inappropriate or irrelevant, not suitable for patients |

**Supplementary Table 2**. The 20 designed questions for question answering.

| 1. Is there any abnormality? |
| --- |
| 1. What is the diagnosis? |
| 1. Where is the abnormality? |
| 1. Is this condition severe? |
| 1. Can this condition cause blindness? |
| 1. Will this condition progress? |
| 1. What is the outcome of this condition? |
| 1. What is the prognosis for this condition? |
| 1. What might be the cause of this condition? |
| 1. What are some common complications that can occur? |
| 1. What should I do? |
| 1. What is your suggestion for me? |
| 1. What kind of further testing might be necessary? |
| 1. What might be the treatment options for this condition? |
| 1. How can I prevent this condition? |
| 1. Can you explain the procedure of a slit lamp exam and what it can detect? |
| 1. How do I prepare for a slit lamp exam? |
| 1. Do I have to have my eyelids rolled up during the examination, and will I get hurt because of this? |
| 1. How long does a slit lamp exam usually take to complete? |
| 1. What are the risks of a slit lamp exam? |
