# Supplementary figures and images for: Slit Lamp Report Generation and Question Answering: Development and Validation of a Multimodal Transformer Model with Large Language Model Integration
Source: J Med Internet Res. 2024 Dec 30;26:e54047. doi: 10.2196/54047 (PMC11729784; doi:10.2196/54047)

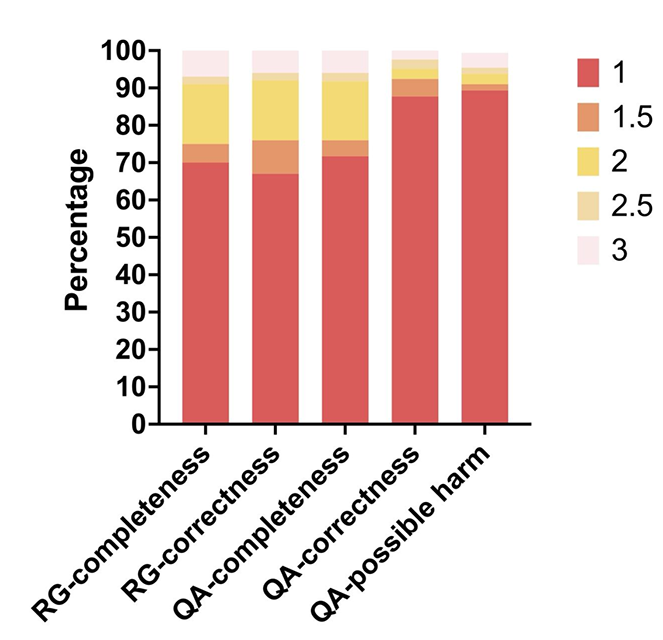

Supplement: Multimedia Appendix 2 [file jmir_v26i1e54047_app2.png]

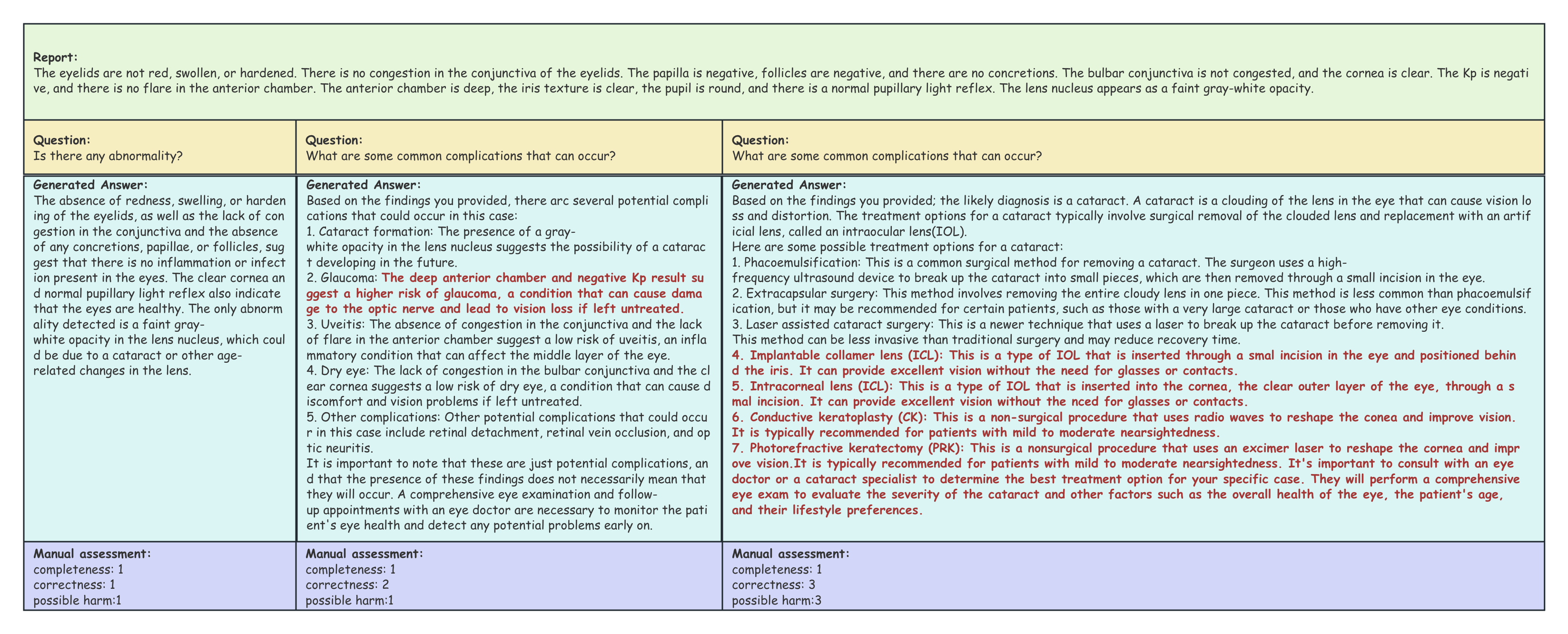

Supplement: Multimedia Appendix 3 [file jmir_v26i1e54047_app3.png]
